# Unraveling the mechanisms of intrinsic drug resistance in *Mycobacterium tuberculosis*

**DOI:** 10.3389/fcimb.2022.997283

**Published:** 2022-10-17

**Authors:** Nicholas C. Poulton, Jeremy M. Rock

**Affiliations:** Laboratory of Host-Pathogen Biology, The Rockefeller University, New York, NY, United States

**Keywords:** tuberculosis, intrinsic resistance, chemical genetics, drug repurposing, drug discovery

## Abstract

Tuberculosis (TB) is among the most difficult infections to treat, requiring several months of multidrug therapy to produce a durable cure. The reasons necessitating long treatment times are complex and multifactorial. However, one major difficulty of treating TB is the resistance of the infecting bacterium, *Mycobacterium tuberculosis* (Mtb), to many distinct classes of antimicrobials. This review will focus on the major gaps in our understanding of intrinsic drug resistance in Mtb and how functional and chemical-genetics can help close those gaps. A better understanding of intrinsic drug resistance will help lay the foundation for strategies to disarm and circumvent these mechanisms to develop more potent antitubercular therapies.

## Introduction

*Mycobacterium tuberculosis* (Mtb) infection is notoriously difficult to treat. Standard treatment regimens for drug sensitive tuberculosis (TB) typically last for 6 months and involve combination therapy with 2-4 antibiotics, depending on the stage of treatment (Dorman et al., 2021; WHO, 2021). Even with 6 months of chemotherapy, 5-10% of patients may experience disease relapse (Lambert et al., 2003; Merle et al., 2014; Colangeli et al., 2018). The difficulty of treating TB can be attributed to a multitude of factors including variable drug penetration into infected lesions (Dartois, 2014; Lenaerts et al., 2015) and treatment lapses due to toxic drug side effects (Tostmann et al., 2008; Seddon et al., 2012; Si et al., 2018; Conradie et al., 2020). However, a major contributor to the difficulty of treating TB is the problem of bacterial drug resistance, which can broadly be classified into two main categories: intrinsic drug resistance and acquired drug resistance (Walker et al., 2015; Xu et al., 2017; Batt et al., 2020). Bacterial drug resistance is phenotypically distinct from drug tolerance and persistence (Brauner et al., 2016; Balaban et al., 2019), which will not be reviewed here.

Intrinsic resistance refers to an innate property of a bacterial species that renders an antibacterial, or group of antibacterials, less effective (Blair et al., 2015; Peterson and Kaur, 2018). Importantly, intrinsic resistance mechanisms are usually present in all (or almost all) members of a bacterial species. In some cases, genes imparting intrinsic resistance appear to have evolved specifically for protection against antibacterial compounds (Madsen et al., 2005; Liu et al., 2019). For example, Mtb encodes *erm(37)*, a 23S rRNA methyltransferase that protects the ribosome from macrolide, lincosamide, and streptogramin binding (Madsen et al., 2005). *erm(37)* does not have a known role in bacterial growth, virulence, or stress tolerance and likely evolved to protect ancestral, soil-dwelling actinobacteria against ribosome-targeting natural products produced by themselves or their neighbors (Morris et al., 2005). In other cases, genes essential for microbial growth and virulence can contribute to intrinsic drug resistance (Tan et al., 2012; Batt et al., 2020; Dulberger et al., 2020). For example, many essential genes in Mtb are involved in cell envelope biosynthesis and regulation. The Mtb cell envelope protects Mtb from host immune pressure and serves as a selective barrier to antibiotic penetration (Johnson et al., 2019; Peterson et al., 2021).

Acquired drug resistance refers to antibiotic resistance that evolves through specific chromosomal mutations or horizontal gene transfer (Peterson and Kaur, 2018; Evans et al., 2020). In Mtb all acquired drug resistance arises as a result of mutation since there is no evidence for recent horizontal gene transfer in Mtb (Boritsch et al., 2016). Many of the mutations that confer high-level acquired drug resistance in Mtb have been well studied and characterized (Walker et al., 2015; CRyPTIC Consortium, 2018; Hunt et al., 2019). For example, partial loss-of-function mutations in the isoniazid (INH)-activating enzyme *katG* are the primary mechanism by which INH resistance emerges (Zhang et al., 1992). Rifampicin resistance emerges primarily through point mutations in the rifampicin resistance determining region on the beta subunit of RNA polymerase (*rpoB*) (Yamada et al., 1985; Telenti et al., 1993). Although many resistance-conferring mutations have been identified over the years, there is a growing appreciation for drug resistance mutations that fall outside the drug activator or target and which typically confer low-to-intermediate resistance (Wong et al., 2011; Colangeli et al., 2018; Hicks et al., 2020). Such low-to-intermediate resistance is clinically relevant (Colangeli et al., 2018) but much more poorly understood. While there remains much to be explored regarding acquired drug resistance in Mtb, this is a topic covered extensively in other reviews (including reviews in this series) and will not be a major focus here.

This review will first briefly outline existing methods used to define intrinsic drug resistance mechanisms in Mtb. We will then review our current understanding and knowledge gaps of intrinsic drug resistance in Mtb and highlight how functional and chemical-genetics (Sassetti et al., 2001; Kim et al., 2013; Bosch et al., 2021) can help close those gaps. We end with a brief discussion on how existing genetic approaches could be expanded to further intrinsic drug resistance research.

## Chemical-genetic approaches to define intrinsic drug resistance mechanisms in Mtb

Chemical-genetic studies have been a pillar of biology for decades. This vast body of literature covers studies of species from all three domains of life and serves as a rich resource for understanding basic biology as well as informing drug discovery efforts (Parsons et al., 2006; Lehár et al., 2008; Nichols et al., 2011; Brown et al., 2014; Cacace et al., 2017; Antonova-Koch et al., 2018). Broadly speaking, chemical-genetics is the study of how genetic alterations influence the activity of a chemical compound. The simplest form of chemical-genetics relies on spontaneous mutagenesis to study the relationships between genes and drugs. In a given population of bacteria, drug resistance mutations can arise spontaneously at a low frequency and can be isolated by plating on a selective antibiotic concentration (Luria and Delbrück, 1943; Jin and Gross, 1988). Genomes of drug-resistant clones can then be sequenced to determine the mutations causing drug resistance. This simple yet elegant approach has been used for decades to identify some of the most common mechanisms of antibiotic resistance.

**Figure 1:**
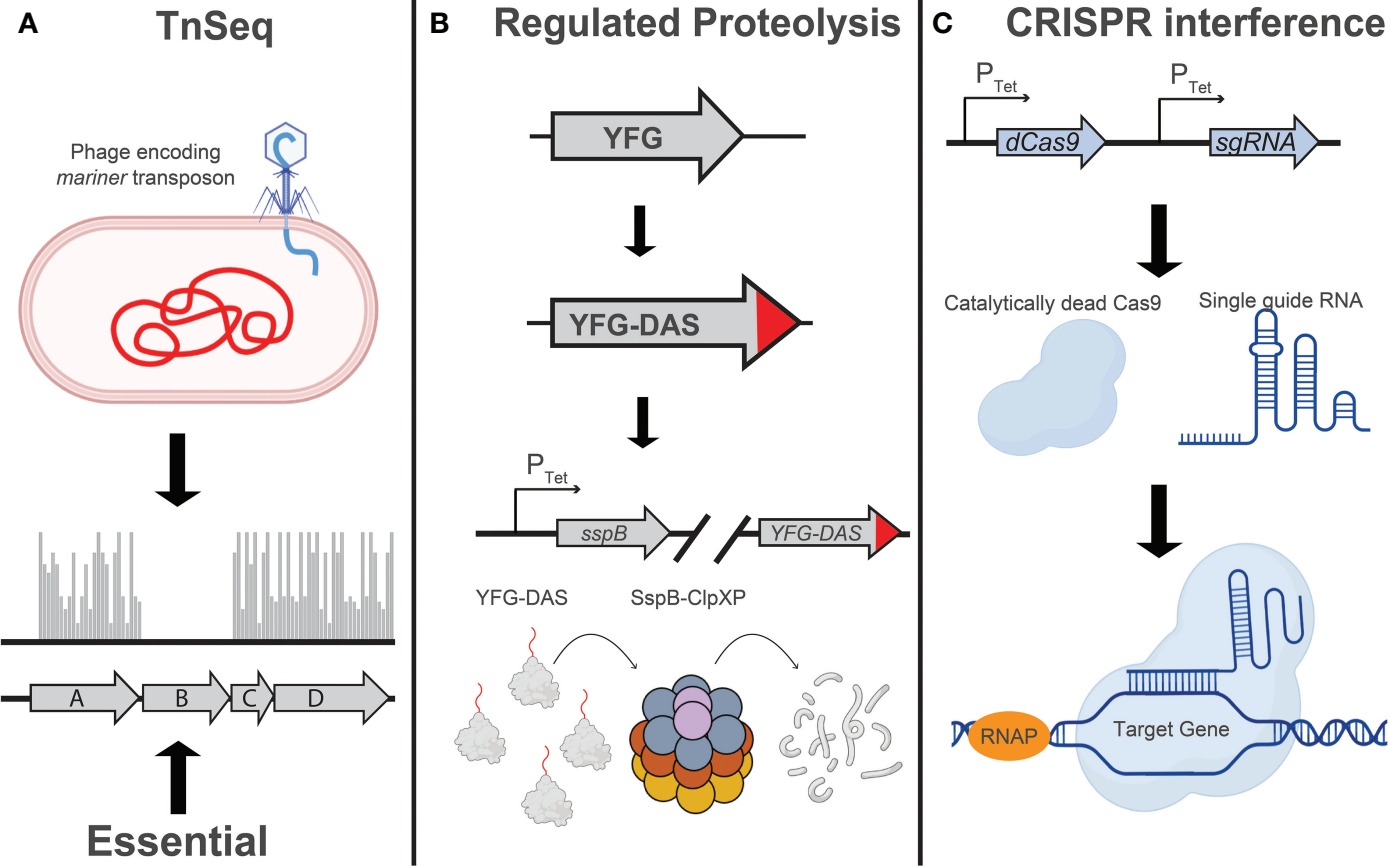
Techniques for large-scale genetic studies in Mtb: Illustration depicting the principles of **(A)** TnSeq, **(B)** regulated proteolysis, and **(C)** CRISPR interference as implemented in mycobacteria. **(A)** Transposon sequencing (TnSeq) involves transduction of Mtb with a phage encoding a mariner transposon, which will randomly insert at TA dinucleotides in the Mtb genome (Sassetti et al., 2001; Dejesus et al., 2017). The absence of transposon insertion events, as determined by next generation sequencing, indicates the essentiality of that gene under the conditions assayed. **(B)** Regulated proteolysis involves the addition of a C-terminal “DAS” tag to an endogenous Mtb gene (Kim et al., 2013; Johnson et al., 2019). Addition or removal of tetracycline, depending on the variant of TetR being used, will induce expression of the proteolytic adaptor, SspB, and facilitate ClpXP-mediated degradation of the DAS-tagged protein. The level of target knockdown can be modulated by using different Tet-regulated promoters to drive *sspB* expression. **(C)** CRISPR interference (CRISPRi) utilizes a single guide RNA (sgRNA) to localize a nuclease-dead Cas9 enzyme to a specific target sequence in the Mtb genome, sterically blocking transcription initiation or elongation. Modulation of target knockdown level can be achieved by designing an sgRNA targeting a divergent protospacer adjacent motif (PAM) sequence, which is directly adjacent to the sgRNA target sequence (Rock et al., 2017), or modulating the extent of complementarity between the sgRNA and the DNA target (Qi et al., 2013; Vigouroux et al., 2018; Hawkins et al., 2020; Bosch et al., 2021; Mathis et al., 2021). Figure graphics were generated using Biorender software.

Other applications of chemical-genetics in Mtb rely on active disruption of target genes (**Figure 1**). One such technique, transposon mutagenesis, involves phage-mediated transduction and integration of a mariner transposon at random TA dinucleotide sequences in the Mtb genome (Sassetti et al., 2001; Dejesus et al., 2017). As typically used in Mtb, this approach results in the irreversible inactivation of target genes and is thus restricted to the analysis of *in vitro* non-essential genes as mutants for *in vitro* essential genes are lost during library construction. Despite the strong GC bias in the Mtb genome, the overwhelming majority of Mtb genes are sufficiently susceptible to transposition for this technique to work efficiently at genome scale (Dejesus et al., 2017). Transposon sequencing (TnSeq) has been used to study chemical-genetic interactions in axenic culture (Xu et al., 2017; Furió et al., 2021; Thiede et al., 2022) as well as in macrophage and mouse models of infection (Bellerose et al., 2020; Kreutzfeldt et al., 2022).

More recently, genetic techniques have been developed that are more applicable to the study of *in vitro* essential Mtb genes. One such technique relies on a regulated proteolysis system, wherein the protein of interest is tagged with a C-terminal “degron” that is recognized by a tetracycline-regulated proteolytic adapter. Upon addition or removal of tetracycline (depending on the variant of the TetR used in the study), the *sspB* adapter is expressed and the corresponding protein is degraded (Kim et al., 2013). In a tour de force, Johnson et al. used a barcoded library of Mtb degron mutants coupled with next generation sequencing to profile over 50,000 compounds to identify target-compound chemical-genetic interactions. The authors identify the putative molecular target for over 40 of these compounds, some of which are active against novel therapeutic targets such as the essential efflux pump *efpA* (Johnson et al., 2019). Throughout the review we will use the term “degron libraries” to refer to this regulated proteolysis technique.

Blending some of the attractive capabilities of both TnSeq and the degron approach, CRISPR interference (CRISPRi) has been used by several labs, including our own, to perform targeted transcriptional inhibition of essential and non-essential genes (Choudhary et al., 2015; de Wet et al., 2020; Bosch et al., 2021; McNeil et al., 2021). This technique leverages the targeting specificity of CRISPR-Cas systems to localize a catalytically dead Cas9 protein to a gene of interest, serving as a steric block to transcription (Qi et al., 2013; Peters et al., 2016). Recently, we have used this system at genome-scale to profile a select group of antitubercular drugs (Li et al., 2022).

## The mycobacterial envelope as a first line of intrinsic antibiotic resistance

It has long been appreciated that mycobacteria have a high level of intrinsic resistance to a diverse set of antibiotics (Jarlier and Nikaido, 1994; Gygli et al., 2017; Xu et al., 2017; Batt et al., 2020; Dulberger et al., 2020). This phenotype has generally been attributed to the relative impermeability of the mycobacterial envelope, which is distinct from those of classic Gram-negative or Gram-positive bacterial species. The Mtb envelope is a complex network composed primarily of peptidoglycan, arabinogalactan, and mycolic acids, which we will refer to as the mAGP complex. At the innermost layer closest to the plasma membrane is the peptidoglycan (PG), which is itself covalently linked to a network of arabinogalactan (AG) polymers (Dulberger et al., 2020). Connected to the AG by esterification is a thick layer of long-chain fatty acids called mycolic acids which form a pseudo-outer membrane bilayer known as the mycobacterial outer membrane (MOM) or mycomembrane (Jankute et al., 2015). Interspersed in the mycolic acids are a select group of proteins including porin-like proteins (Siroy et al., 2008; Wang et al., 2020) and secretion systems (Ates et al., 2015; Tiwari et al., 2020), as well as virulence associated glycolipids such as phthiocerol dimycocerosates (PDIMs) (Rens et al., 2021). Outside of the MOM is a mycobacterial capsule which is composed primarily of complex carbohydrates such as α-D-glucan and D-arabino-D-mannan, but also a select group of lipids and proteins (Stokes et al., 2004; Kalscheuer et al., 2019). Due to the typical growth conditions used in mycobacterial media, the capsule is generally stripped from the Mtb cell surface and is not studied during axenic growth (Stokes et al., 2004). The implications of this fact will be discussed later in this review.

The intrinsic resistance of Mtb to many different classes of antibiotics is often attributed to the impermeability of the MOM (Jarlier and Nikaido, 1994; Batt et al., 2020). Hydrophilic solutes are unable to traverse the mycolic acids and are thought to rely on protein-mediated translocation *via* porin-like proteins (Jarlier and Nikaido, 1994; Ates et al., 2015; Batt et al., 2020; Wang et al., 2020). Hydrophobic compounds are thought to get stuck in a poorly fluid mycolic acid sink and fail to efficiently traverse the MOM. There are substantial data to support this model using both chemical and genetic disruption of the mycolic acid network to potentiate antibiotic uptake and activity (Liu and Nikaido, 1999; Larrouy-Maumus et al., 2016; Xu et al., 2017). The clinical implications of this phenomenon can be seen by the synergistic interaction between rifampicin, which inhibits RNA polymerase (Campbell et al., 2001), and ethambutol, which inhibits arabinogalactan and lipoarabinomannan (LAM) biosynthesis (Goude et al., 2009; McNeil et al., 2019; Zhang et al., 2020). Because arabinogalactan serves as an anchor for the mycolic acid layer, AG inhibitors like ethambutol also perturb the MOM (Kilburn and Takayama, 1981; Mikusová et al., 1995; Dulberger et al., 2020). Despite its relatively modest *in vitro* and *in vivo* activity, ethambutol is included as part of the first-line RIPE (**r** ifampicin, **i** soniazid, **p** yrazinamide, **e** thambutol) regimen for drug sensitive TB. It has previously been suggested that ethambutol mainly serves as a “safety net” to prevent the emergence of rifampicin and isoniazid resistant TB (Dubé et al., 1997). More recently, the clinical importance of ethambutol has been attributed to the efficient distribution of this drug throughout TB lung lesions (Zimmerman et al., 2017). In addition to these roles, ethambutol’s clinical success may be due to its synergistic interaction with rifampicin (Cokol et al., 2017; McNeil et al., 2019). Rifampicin is a hydrophobic, high molecular weight compound for which the mycobacterial envelope serves as a permeability barrier (Xu et al., 2017; McNeil et al., 2019; Li et al., 2022). By disrupting proper formation of arabinogalactan, ethambutol promotes more efficient uptake of rifampicin to exert its bactericidal effect (Cokol et al., 2017; McNeil et al., 2019).

Despite the long-standing appreciation that bacterial surface structures can impede antibiotic uptake, the physiochemical details of this phenomenon are not fully understood in mycobacteria. Chemical-genetic studies have shown that mAGP-related mutants in Mtb are hypersusceptible to certain antibiotics but not others, suggesting that the cell envelope is a relevant barrier for certain drugs such as rifampicin and bedaquiline, but not other drugs like linezolid (Davis et al., 2014; Larrouy-Maumus et al., 2016; Li et al., 2022). In the latter scenario it is unclear whether there are specific importers for these drugs (Rempel et al., 2020; Li et al., 2022) or whether drug diffusion is unaffected by the envelope. While compound size certainly seems to negatively affect uptake beyond a certain threshold (Davis et al., 2014; Li et al., 2022), the physiochemical properties that allow some compounds but not others to easily traverse the Mtb envelope are not fully established. Future chemical-genetic studies could be focused on profiling mAGP-associated mutants against a physiochemically diverse set of antitubercular compounds, or in practice any compounds for which uptake can be quantitatively monitored, to identify which chemical scaffolds are efficiently blocked by the Mtb envelope (Davis et al., 2014; Zgurskaya and Rybenkov, 2019). This approach can be achieved using existing genome-scale CRISPRi libraries (Bosch et al., 2021). Alternatively, more targeted libraries (degron or CRISPRi) can be generated to specifically target mAGP-associated genes and profile the susceptibilities of each mutant. These screens could help reveal which physiochemical properties are associated with the ability or inability to traverse the mycobacterial envelope and help to define the “rules” of drug uptake in mycobacteria (Davis et al., 2014; Larrouy-Maumus et al., 2016; Zgurskaya and Rybenkov, 2019; Zhao et al., 2020).

At the same time, such screens could also reveal how different molecular structures within the envelope serve as a barrier to antibiotic uptake. Although often viewed as a unitary structure, the mAGP network is remarkably complex and disrupting different components of this structure may differentially sensitize Mtb to particular compounds. For example, knockdown of many arabinogalactan and mycolic acid biosynthetic enzymes seems to potentiate the activity of bedaquiline (Lupien et al., 2018; Li et al., 2022). However, for reasons that remain unclear, this effect is not observed as strongly with disruption of peptidoglycan biosynthetic enzymes (Li et al., 2022). Are these differential phenotypes simply a result of genetic redundancy in peptidoglycan biosynthesis, or do they reflect some degree of barrier specificity for different envelope structures?

While chemical-genetic interactions can help inform which physiochemical properties and potentially which envelope structures are most important for intrinsic drug resistance, there are several limitations. For example, many antitubercular compounds target envelope biosynthesis either directly (Banerjee et al., 1994; Goude et al., 2009) or indirectly (Stover et al., 2000; Thiede et al., 2022). Let’s assume that a CRISPRi knockdown strain against the essential arabinogalactan biosynthetic enzyme *dprE1* renders Mtb more sensitive to a given compound. There are several potential explanations to explain this interaction. First, DprE1 or another target involved in arabinogalactan biosynthesis may be the direct target of the screened compound (Kumar et al., 2018). Second, lack of *dprE1* activity may weaken the arabinogalactan layer sufficiently to increase envelope permeability and compound uptake. Third, the chemical-genetic interaction may be independent of compound uptake and reflect a more mechanism-specific collateral vulnerability associated with *dprE1* inhibition and arabinogalactan biosynthesis perturbation (Wang et al., 2019). Therefore, care should be exercised when interpreting chemical-genetic interactions and such studies should be coupled with mass spectrometry drug-uptake quantification to differentiate between these various possibilities (Davis et al., 2014; Planck and Rhee, 2021). Further, structural and/or biochemical approaches can be used to identify the target of a particular compound, helping to differentiate between a direct or an indirect mechanism for a specific chemical-genetic interaction (Pellecchia et al., 2002; Zhang et al., 2020; Ottavi et al., 2022).

Moreover, growth of Mtb in axenic culture often ignores two key components of the mycobacterial cell surface. PDIMs are a family of lipids involved in Mtb virulence, with over 1% of the Mtb genome dedicated to PDIM biosynthetic genes (Trivedi et al., 2005; Domenech and Reed, 2009; Rens et al., 2021). Because of the metabolic costs associated with synthesizing PDIMs and the fact that these lipids are not only dispensable in standard axenic culture but can restrict permeability of culture carbon sources (Ates et al., 2015; Wang et al., 2020), lab-grown Mtb frequently sustains loss of function mutations in PDIM biosynthetic enzymes (Domenech and Reed, 2009). The lack of PDIMs has been associated with increased sensitivity to drugs and altered nutrient uptake, consistent with PDIMs being a relevant permeability barrier in Mtb (Soetaert et al., 2015; Wang et al., 2020). Therefore, care should be taken to assess the role of PDIM in compound uptake. Lastly, mycobacteria are frequently cultured in the presence of detergent to prevent cell clumping. Detergents act by stripping the mycobacterial capsule, which may influence Mtb’s small molecule permeability (Stokes et al., 2004; Kalscheuer et al., 2019). Therefore, as with PDIM, confirmation of relevant chemical-genetic interactions should be performed under conditions in which the Mtb capsule is intact (e.g. infection models, detergent-free plates, etc.).

The Mtb envelope is not a static structure and is influenced by the growth environment of the bacteria (Sarathy et al., 2013; Larrouy-Maumus et al., 2016; Koh et al., 2022). For example, Sarathy et al. demonstrated that non-replicating and nutrient-starved bacteria display greatly reduced drug uptake, likely through a cell wall remodeling process that is not entirely understood (Cunningham and Spreadbury, 1998; Wu et al., 2016). Perturbing envelope integrity may play a greater role in increasing compound uptake in Mtb grown under these conditions than standard replicating conditions (Sarathy et al., 2013). Further, Koh et al. showed that rifampicin is less effective when Mtb is grown on the *in vivo-*relevant carbon source cholesterol as a result of modifications to the Mtb envelope (Koh et al., 2022). This effect could be specifically reversed through selective cell envelope disruption. These studies highlight the importance of performing chemical-genetic screens in host-relevant carbon sources and stress conditions. This will help identify environments in which successful cell envelope disruption will facilitate antibiotic entry.

Having a more complete understanding of the mycobacterial envelope as a barrier to antibiotic uptake will pave the way for several important applications. First, this knowledge can be used to better predict synergistic drug combinations and inform the preclinical testing of new combination therapies. For example, several studies have shown that bedaquiline can be potentiated by inhibiting proper mAGP synthesis (Lechartier et al., 2012; Lupien et al., 2018; Li et al., 2022). Part of the success of the bedaquiline, pretomanid, and linezolid (BPaL) combination (Conradie et al., 2020) may be due to the disruption of mycolic acids by pretomanid (Stover et al., 2000; Manjunatha et al., 2009), resulting in increased bedaquiline uptake. This synergy would likely extend to pre-clinical DprE1 inhibitors (Lechartier et al., 2012; Lupien et al., 2018). Second, knowledge of the genetic regulation of cell envelope synthesis will allow for the rational prioritization of target-based drug discovery candidates. Ideally, these studies will identify targets for which inhibition not only leads to bacterial death but also potentiates the uptake and activity of other drugs. Third, as mentioned above, knowledge of the physiochemical “rules” that allow compounds to traverse the mAGP will help direct medicinal chemistry efforts to improve compound uptake. Lastly, this knowledge can be used to identify mechanism-specific synergies that target different components of the mAGP complex. Isoniazid and ethambutol have been used together in first-line TB therapy for decades but have been shown to be slightly antagonistic or additive at best (Cokol et al., 2017; Larkins-Ford et al., 2021). Chemical-genetic profiling of cell envelope-targeting compounds may help to reveal other targets, either in the same pathway or parallel pathways, that will act synergistically, thus optimizing the therapeutic potential of this highly vulnerable chemical complex.

## Efflux pumps as the next line of defense against antibiotics

The selective permeability of the mycobacterial envelope collaborates with additional mechanisms to promote intrinsic drug resistance (Nikaido, 1994). Once a chemical compound traverses the mAGP, it can encounter another line of defense in the form of drug efflux pumps (Piddock et al., 2000; Davis et al., 2014; Laws et al., 2022). Efflux pumps are transmembrane proteins that facilitate the transport of small molecules out of the periplasm and/or the cytosol (Blair et al., 2015; Venter et al., 2015; Boyer et al., 2022). Mtb encodes several dozen putative and validated drug efflux pumps, which have been comprehensively reviewed by Laws et al. (Laws et al., 2022). Some efflux pumps, such as those of the ATP Binding Cassette (ABC) family, are regulated by ATP hydrolysis (Braibant et al., 2000; Pasca et al., 2004), whereas major facilitator superfamily (MFS) efflux pumps are regulated by proton-induced conformational changes (Li et al., 2017; Laws et al., 2022). Other pumps, such as those of the resistance-nodulation-cell-division (RND) superfamily rely on a drug-proton antiporter mechanism (Venter et al., 2015; Li et al., 2017; Laws et al., 2022). The efflux pumps of Mtb vary greatly in their compound specificity with some having a single validated transported substrate and others having many substrates (Liu et al., 2019).

The clinical importance of drug efflux in Mtb has been well established. For example, the MmpS5/L5 efflux pump (RND superfamily) has been shown to be active against several drugs including bedaquiline and clofazimine (Hartkoorn et al., 2014; Briffotaux et al., 2017). Expression of the *mmpS5/L5* operon is negatively regulated by the transcriptional repressor MmpR (Rv0678) (Briffotaux et al., 2017). Loss of function mutations in *rv0678* result in constitutive MmpS5/L5 expression and confer acquired drug resistance to bedaquiline, representing a significant complication to the long-term success of this new TB drug (de Vos et al., 2019; Nimmo et al., 2020). Interestingly, for reasons that remain unclear, some clinical Mtb strains harbor loss-of-function *rv0678* mutations that pre-date the clinical use of bedaquiline (Villellas et al., 2017). The presence of these mutations could reflect earlier clinical exposure to clofazimine or other drugs. Another example is Rv1258c (Tap), an MFS efflux pump active against several antituberculars including streptomycin and rifampicin (Adams et al., 2011; Adams et al., 2019; Liu et al., 2019). Tap expression is activated by the transcription factor *whiB7*. WhiB7 or genes involved in its regulation can in turn sustain mutations that result in constitutive activation of the WhiB7 regulon, including Tap, to promote acquired drug resistance (Reeves et al., 2013; Schrader et al., 2021; Li et al., 2022). Thus, efflux pumps like MmpS5/L5 and Tap promote intrinsic resistance in Mtb and can further be augmented by mutation to promote acquired drug resistance.

Most validated or putative efflux pumps in Mtb are poorly characterized, but it is likely that efflux pumps beyond MmpS5/L5 and Tap contribute to intrinsic drug resistance in Mtb (Remm et al., 2022). An in-depth characterization of these under-studied efflux pumps is much needed (Szumowski et al., 2012). To facilitate this characterization, one could systematically generate underexpression and overexpression strains for all predicted Mtb efflux pumps. For example, a small, targeted CRISPRi library (Bosch et al., 2021) could be generated that contains knockdown strains for all validated and predicted efflux pumps. This library could be treated with a wide range of antitubercular compounds to determine which mutants display reduced fitness under which drug treatment conditions. Some efflux pumps may overlap in the types of compounds transported (Smith and Blair, 2014). To address this possibility, combinatorial libraries in which multiple efflux pump genes are simultaneously silenced (Wong and Rock, 2021; Li et al., 2022) may help to identify functional redundancies between efflux pumps. Further, because some efflux pumps may not be highly expressed under standard lab conditions (Gupta et al., 2010; Adams et al., 2011), knocking down the corresponding gene may not produce a phenotype. To overcome this, a parallel pool of barcoded overexpression strains for each efflux pump could be generated and screened against the same panel of antitubercular compounds (Hicks et al., 2018). Lastly, as in the case for MmpS5/L5 and Tap, one could interrogate the increasing amount of Mtb clinical strain genome sequencing available to identify predicted efflux pumps or their regulators under positive selection. Should such evidence exist, it seems reasonable to predict that the relevant selective pressure is antibiotics, although other mechanisms cannot be ruled out.

In designing these experiments, it will be important to carefully curate the list of predicted efflux pumps. For example, several ABC proteins have been annotated as efflux pumps even though these proteins lack transmembrane helices (Duan et al., 2019; Laws et al., 2022). Two such previously annotated efflux pumps, Rv1473 and EttA, turn out to indeed influence drug activity in Mtb but have nothing to do with efflux and rather are ATP-dependent regulators of the ribosome (Sharkey et al., 2016; Cui et al., 2022; Li et al., 2022). Furthermore, genetic studies of efflux pumps should be validated with biochemical approaches to unambiguously demonstrate drug efflux activity.

Given the role of drug efflux in intrinsic and acquired Mtb drug resistance, there has been considerable interest in developing efflux pump inhibitors (EPIs) to potentiate TB drug regimens. Numerous small molecule EPIs, including both natural products and synthetic compounds, have been described (Szumowski et al., 2012; Machado et al., 2016; Laws et al., 2022). Many of these EPIs show broad activity against numerous efflux pumps and appear to act in a relatively non-specific manner by disrupting membrane energetics (Adams et al., 2014; Ruth et al., 2020; Laws et al., 2022). Indeed, many EPIs have antimycobacterial activity as single agents, which could reflect synthetic lethality of multi-efflux pump inhibition or an efflux pump independent mode of action, e.g. disruption of membrane energetics (Chen et al., 2018; Remm et al., 2022). The pleiotropic consequence of disrupting membrane energetics may confound the interpretation how EPIs potentiate the activity of other TB drugs (Amaral and Viveiros, 2017; Chen et al., 2018). Despite their unclear mode of action, some EPIs may have potential for use in TB therapy. For example, there are several studies showing that the antipsychotic drug thioridazine has direct antitubercular activity (Amaral et al., 2001; Abbate et al., 2012; Pieroni et al., 2015). Thioridazine also displays synergy with other drugs, possibly by altering membrane potential and reducing drug efflux (Coelho et al., 2015; Machado et al., 2016; Ruth et al., 2020). Although there is no evidence of direct efflux pump inhibition by thioridazine, its ability to disrupt membrane potential may lower cellular ATP levels, thereby limiting the activity of ATP-dependent efflux pumps. Alternatively, or in addition, thioridazine-mediated disruption of proton gradients may alter the ability of MFS efflux pumps to undergo proton-induced conformational changes or of RND family efflux pumps to carry out drug-proton antiport exchange.

Ultimately, while non-specific EPIs may have clinical value, specific and selective EPIs could help augment TB treatment. However, substantial advances in our understanding of efflux pump specificity and structure may be required to identify such compounds. Until then, more generic EPIs, especially those that have stand-alone antitubercular activity, may be of utility for TB treatment (Abbate et al., 2012; Rodrigues et al., 2020).

## Beyond drug uptake and efflux: Cytosolic mechanisms of intrinsic antibiotic resistance

**Figure 2:**
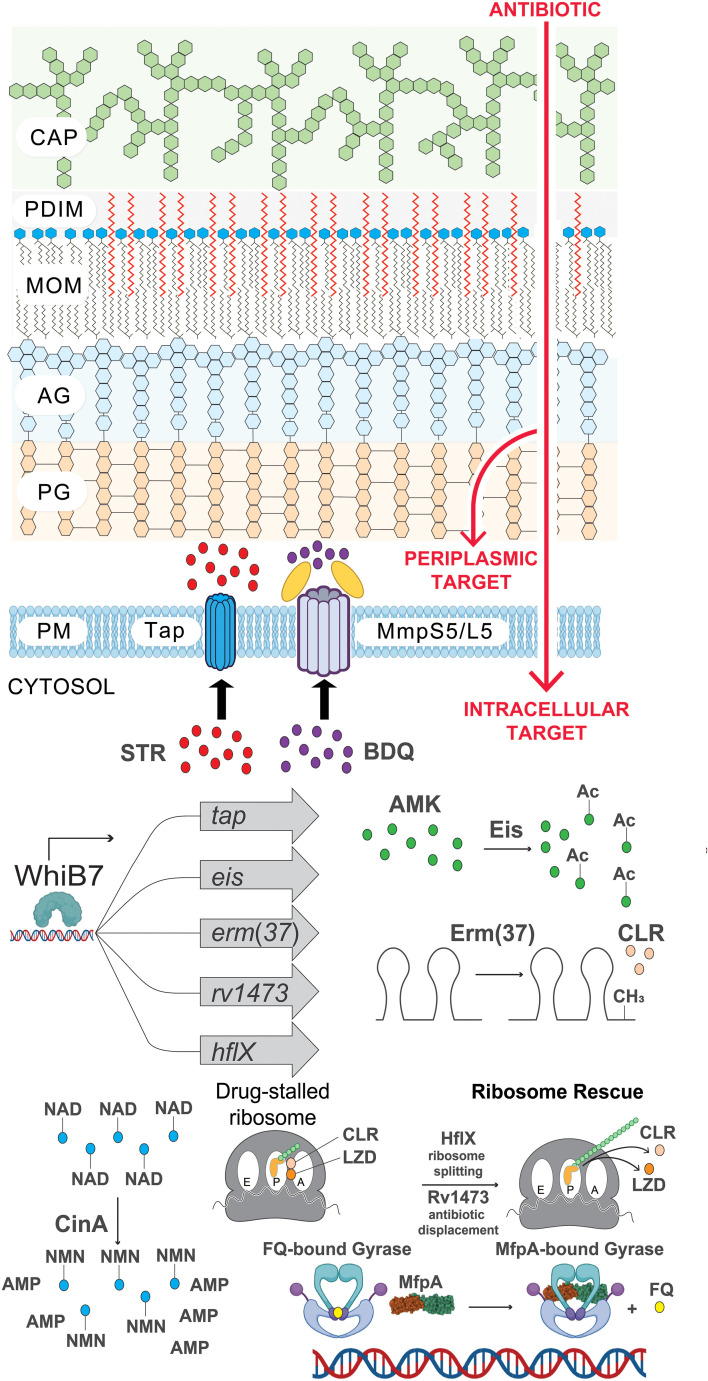
The many layers of intrinsic antibiotic resistance in Mtb. Illustration of intrinsic resistance factors at the Mtb cell surface and inside the cytosol. CAP, capsule; PDIM, phthiocerol dimycocerosates; MOM, mycobacterial outer membrane; AG, arabinogalactan; PG, peptidoglycan; PM, plasma membrane; STR, streptomycin; BDQ, bedaquiline; AMK, amikacin; Ac, acetylation modification (CH_3_CO); CLR, clarithromycin; CH_3_, methylation of ribosomal RNA; LZD, linezolid; FQ, fluoroquinolone; NAD, nicotinamide adenine dinucleotide (depicted as a drug adduct); NMN, nicotinamide mononucleotide; AMP, adenosine monophosphate. Figure graphics were generated using Biorender software.

An intrepid compound has traversed the mycobacterial envelope, avoided efflux, and is ready to engage its target. What next? Once again, Mtb is well-equipped with numerous cytosolic mechanisms of intrinsic drug resistance. Once again, Mtb is well-equipped with numerous cytosolic mechanisms of intrinsic drug resistance (**Figure 2**). (Blair et al., 2015). Not surprisingly, these processes tend to be more drug-specific than selective envelope permeability and efflux, and based on current knowledge are most frequently seen with the antituberculars which target the ribosome (Wilson, 2014). Some of the most well-studied mechanisms of cytosolic intrinsic drug resistance in Mtb are listed in **Table 1**.

**Table 1:** Cytosolic intrinsic drug resistance factors of Mtb.

**Resistance Gene**	**Protection specificity**	**Mechanism**	**Reference**
*whiB7* (*rv3197A*)	Ribosome-targeting antibiotics	Transcription of other resistance factors	(Morris et al., 2005)
*erm(37)* (*rv1988*)	Macrolides, lincosamides, streptogramin B antibiotics	Methylation of the 23S rRNA drug binding site	(Madsen et al., 2005)
*eis* (*rv2416c*)	Amikacin and kanamycin	Aminoglycoisde acetylation and inactivation	(Zaunbrecher et al., 2009)
*hflX* (*rv2725c*)	Macrolides, lincosamides	Rescue of stalled ribosomes	(Rudra et al., 2020)
*ocrA (rv1473)*	Oxazolidinones and phenicols	Drug displacement from ribosome	(Sharkey et al., 2016; Antonelli et al., 2018; Li et al., 2022)
*smpB/ssr* (*rv3100c/ssr*)	Oxazolidinones, phenicols, clarithromycin	Rescue of stalled ribosomes	(Li et al., 2022)
*mfpAB* (*rv3361c/rv3362c*)	Fluoroquinolones	DNA mimicry, protection of DNA gyrase from FQs	(Hegde et al., 2005; Tao et al., 2013)
*cinA* (*rv1901*)	Isoniazid, ethionamide, nitroimidazoles	Cleavage of drug-NAD adducts	(Wang et al., 2011; Kreutzfeldt et al., 2022)

The different layers of intrinsic cytosolic resistance can all be seen within the *whiB7* pathway. WhiB7 is a transcription factor that senses translational stalling which can be triggered by ribosome stress during drug treatment, host-derived stressors, and poorly characterized metabolic changes (Morris et al., 2005; Burian et al., 2013). During unstressed conditions, *whiB7* expression is low due to upstream ORF (uORF)-mediated transcription attenuation (Lee et al., 2022). Translation of the uORF in the *whiB7* 5’ leader fails to prevent formation of a Rho-independent terminator, resulting in transcription termination prior to transcription of the *whiB7* ORF. However, stalled translation of the uORF promotes formation of an antiterminator, resulting in high-level transcription of the *whiB7* ORF. This subsequently further activates transcription from the *whiB7* promoter and those of the WhiB7 regulon genes. Among the WhiB7 regulon is *tap*, the multidrug efflux pump described in the previous section. Furthermore, WhiB7 promotes the transcription of several cytosolic resistance factors including *eis*, an aminoglycoside acetyltransferase that can chemically modify and inactivate amikacin and kanamycin (drug modification) (Zaunbrecher et al., 2009). Another WhiB7 regulon gene is *erm(37)*, a ribosomal RNA methyltransferase which modifies the macrolide binding site on the 23S rRNA to prevent drug binding (target modification) (Madsen et al., 2005). Moreover, WhiB7 promotes transcription of *hflX*, a ribosome recycling factor that can help to rescue stalled ribosomes (target rescue) (Rudra et al., 2020). This WhiB7 pathway presumably evolved in an ancestral soil-dwelling actinobacterium that encountered ribosome-targeting antibiotics in its environment.

## Expanding our knowledge of cytosolic intrinsic resistance factors & how to overcome them

Compared to the hundreds of genes which contribute to intrinsic resistance by regulating cell envelope processes (Xu et al., 2017; Li et al., 2022), there are many fewer known instances of resistance factors in the Mtb cytosol. This can likely be explained by the fact that cell envelope-associated intrinsic resistance factors are likely to be pleiotropic by preventing the uptake of many diverse compounds, whereas cytosolic resistance factors are likely to be specific to a particular drug or class of drugs. The relative paucity of known cytosolic resistance factors may also be explained by the limited scope of the drugs that have been screened in chemical-genetic studies. Future chemical-genetic screening efforts could focus on comprehensively defining the intrinsic “resistome” for a larger panel of antitubercular drugs.

We posit that there is merit to performing chemical-genetic profiling on FDA approved drugs with detectable but limited antitubercular activity. Although in some cases this lack of potency may be explained by poor drug uptake (which could potentially be improved by mAGP disruption) or alteration of a specific molecular target, in some cases it may be the result of a specific intrinsic resistance factor. This is the case with macrolides which are ineffective against Mtb due to *whiB7*-mediated expression of *erm(37)* (Morris et al., 2005). In instances where *whiB7* has been mutationally inactivated, clarithromycin displays potent activity against Mtb (Warit et al., 2015; Li et al., 2022). Using the macrolide paradigm, where *whiB7* is central to Mtb’s intrinsic resistance, there may be parallel cases where one or several factors are responsible for limiting the activity of a given drug. Ultimately, by defining the intrinsic “resistome” for these compounds it may allow for three potential follow up strategies to advance their potential use in the clinic.

One such strategy involves leveraging “acquired drug sensitivities.” Here, clinically prevalent mutations in intrinsic resistance factors may present therapeutic opportunities for drugs that are already approved for clinical uses outside of TB treatment. As described above, there are cases where Mtb clinical isolates sustain loss-of-function mutations in intrinsic resistance genes. In addition to the loss-of-function mutations in *whiB7*, there are several documented loss-of-function mutations in the *mmpS5/L5* efflux pump (Merker et al., 2020; Li et al., 2022). These mutations render Mtb hypersusceptible to bedaquiline and clofazimine. As the use of whole genome sequencing is expanded in clinical labs, we may be able to predict unique drug susceptibilities based on the genome sequence of the infecting strain (CRyPTIC Consortium, 2018; Doyle et al., 2018). This may be particularly useful for multidrug-resistant and extensively-drug resistant TB cases with limited treatment options. Until “personalized” TB treatment is more widely available, geographically concentrated sublineages may be targeted on the basis of their unique vulnerability to particular drugs (Phelan et al., 2019; Li et al., 2022).

Another strategy to potentiate antibiotic activity against Mtb is to specifically inhibit intrinsic resistance pathways. Developing small molecule inhibitors of intrinsic resistance factors may synergize with the drug of interest (Hugonnet et al., 2009; Kurz et al., 2016). The classic example of this can be seen with the use of beta lactamase inhibitors to prevent the degradation of beta lactam antibiotics. In addition to the Mtb beta lactamase, BlaC, two other well-characterized examples which modify drugs or drug adducts are Eis and CinA (Zaunbrecher et al., 2009; Kreutzfeldt et al., 2022). Many more drug-modifying enzymes likely remain to be discovered (Zaunbrecher et al., 2009; Kreutzfeldt et al., 2022). Small molecule discovery efforts could focus on identifying inhibitors of particular drug modifying enzymes. As such, inhibitors of Eis would likely potentiate aminoglycoside activity whereas inhibition of CinA would likely potentiate isoniazid and pretomanid activity. However, both *eis* and *cinA* are non-essential for Mtb growth under standard conditions and inhibitors of these enzymes may face difficulties in preclinical development because in monotherapy, they would be unlikely to have any antimycobacterial activity. Therefore, genetic strategies focusing on essential genes are ideal (degron libraries, CRISPRi) since these represent some of the most attractive drug targets. Essential genes that impart intrinsic drug resistance could be prioritized for target-based drug discovery in order to form synergistic drug combinations where both compounds have individual activity but work more efficiently in combination. One such target is the mycobacterial superoxide dismutase (*sodA*), an essential oxygen radical quenching enzyme. Our previous work identified that knockdown of *sodA* sensitizes Mtb to several different classes of drugs (Li et al., 2022). Inhibitors of *sodA* would likely synergize with other drugs while also producing a direct, antimycobacterial effect.

Finally, a comprehensive understanding of the Mtb intrinsic resistome may allow for the rational design of drug analogs that avoid specific intrinsic resistance mechanisms. There are several examples of this concept, where derivatives of a particular drug are recalcitrant to the resistance mechanisms that target the parent compound. For example, 3^rd^ generation tetracyclines avoid the drug-displacing activity of TetM in certain Gram-positive species (Jenner et al., 2013). Similarly, ketolides are a family of macrolide derivatives that have been engineered to bind Erm-methylated bacterial ribosomes (Capobianco et al., 2000). The ketolide drug telithromycin is approved for the treatment of erythromycin-resistant *S. pneumoniae* infections (Lonks and Goldmann, 2005). This concept is further exemplified by the aminoglycosides in Mtb. Despite being structurally and chemically similar, the aminoglycoside acetyltransferase Eis seems to have activity against kanamycin and amikacin but not streptomycin (Zaunbrecher et al., 2009). Conversely, the drug efflux pump Tap is active against streptomycin but not amikacin (Liu et al., 2019; Li et al., 2022). Another example from Mtb is seen with the beta lactamase BlaC. BlaC efficiently degrades penicillins and cephalosporins, which likely explains the poor activity of these beta lactams against Mtb (Hugonnet et al., 2009; Kurz et al., 2016). However, carbapenem antibiotics such as meropenem are relatively recalcitrant to BlaC activity. Accordingly, meropenem has strong activity against Mtb and has shown promising results in early clinical trials (Diacon et al., 2016). Although meropenem is still paired with a beta lactamase inhibitor, the poor activity of BlaC in degrading meropenem likely explains its superior activity relative to other beta lactams. Although there are only a handful of published drug modifying enzymes in Mtb, this phenomenon is likely to be more common than is currently appreciated (Warrier et al., 2016; Luthra et al., 2018). More expansive metabolomic studies may help identify the modifications made to antibiotics within the Mtb periplasm and cytosol. A better understanding of intrinsic resistance mechanisms coupled with advances in structural biology and docking algorithms (Pagadala et al., 2017; Jumper et al., 2021) may facilitate the design highly specific drug analogs that circumvent the activity of particular intrinsic resistance proteins.

Although a more chemically comprehensive screening effort may reveal unique and novel mechanisms of intrinsic drug resistance, there are some potential pitfalls to this approach. This chemical-genetics strategy would almost certainly fail for a drug like fosfomycin, a non-TB antibiotic which targets the peptidoglycan synthetic enzyme MurA. The lack of fosfomycin activity against Mtb is due to lack of conservation in the drug binding site, not the presence of a specific intrinsic resistance factor (de Smet et al., 1999). Compounds chosen to undergo chemical-genetic profiling should be rationally selected on the basis of target conservation if that information is available. Finally, we believe this strategy of chemical-genetic profiling is important for compounds early in pre-clinical development, especially those with poorly understood mechanisms. Defining the intrinsic resistome may provide insights regarding the molecular target of the compound and also its mode of action (i.e., downstream effects of activity). Following the logic described above, lead compounds may be optimized further through careful pairing in synergistic drug combinations and engineering to avoid the activity of specific resistance factors.

## New genetic tools for chemical-genetic studies

So far we have primarily discussed chemical-genetic studies employing three main genetic techniques: transposon mutagenesis, regulated proteolysis, and CRISPRi. High-density transposon mutagenesis was first applied to Mtb almost two decades ago and it continues to be a rich genetic resource. Regulated proteolysis systems and CRISPRi are relatively new genetic tools for Mtb and present powerful strategies to investigate the role of essential genes. However, all three strategies have technical limitations. Continued innovation in mycobacterial genetics will be important to address some of the gaps in our knowledge of Mtb biology, especially intrinsic drug resistance. As mentioned above, many Mtb genes are not expressed at high levels during standard laboratory culture and thus loss-of-function genetic approaches may be insufficient to reveal a phenotype (Schnappinger et al., 2003; Adams et al., 2011). For example, genetic disruption of a macrophage-induced efflux pump may not sensitize Mtb to antibiotics in broth since it has a low level of expression under those conditions. However, a drug sensitivity phenotype would likely be observed in drug-treated macrophages. These sorts of chemical-genetic interactions could potentially be captured by screening drugs in complex environments that best mimic host-relevant conditions.

Alternatively, overexpression of that efflux pump in broth conditions would likely confer drug resistance. Gain-of-function genetics represents a complementary tool to capture chemical-genetic phenotypes that would be difficult to observe with loss-of-function techniques. However, high throughput mechanisms of gene activation do not yet exist in Mtb. Such techniques have been employed quite successfully in mammalian systems (Ho et al., 2020) and, with a lesser degree of success in other bacterial species (Ho et al., 2020; Kiattisewee et al., 2021). CRISPR activation (CRISPRa) is one such strategy in which a catalytically dead Cas9 is fused to a transcriptional activator (Ho et al., 2020). A specific guide RNA can be used to locate the transcription activating Cas9 to the promoter region of a specific gene or operon. Another option for systematic gene activation in bacteria is to use a transposon carrying a strong, outward-facing promoter (Coe et al., 2019). This system is less specific than CRISPRa and is confounded in many cases by simultaneous activation of one gene and disruption of an adjacent gene. Alternatively, with advances in DNA synthesis, barcoded overexpression plasmids could be built for individual Mtb genes or operons to systematically overexpress Mtb genes. If any of these techniques were employed successfully in Mtb, it may allow for the identification of chemical-genetic phenotypes missed by loss-of-function genetics, particularly for lowly expressed genes.

Lastly, new genetic techniques will be useful for chemical-genetic screens that rely on bacterial outgrowth. For example, Kreutzfeldt et al. (Kreutzfeldt et al., 2022) performed a TnSeq screen to identify mutants with reduced survival in isoniazid-treated macrophages, which involved outgrowth of the surviving bacteria on agar plates. Transposon mutagenesis is well suited for assays that involve bacterial outgrowth since it generally results in irreversible target gene disruption and does not require an “off-switch.” However, both CRISPRi and regulated proteolysis will likely have residual target knockdown after the removal of tetracycline, which may prevent or delay viable mutants for essential genes from successfully resuming growth (Qi et al., 2013). As such, these techniques may not be ideally suited, at least as currently implemented, for these types of screens. Therefore, next generation derivatives of both of these strategies that allow for an efficient “off-switch” will be paramount to screens relying on outgrowth such as those seeking to identify mutants with impaired survival during drug treatment.

## Exploring intrinsic resistance heterogeneity across diverse Mtb strains

*Mycobacterium tuberculosis* displays a remarkable degree of genetic conservation across its major lineages and sublineages (Kremer et al., 1999). This is likely due to the recent evolutionary emergence of Mtb as well as the lack of horizontal gene transfer (Boritsch et al., 2016). However, there is a growing appreciation that the genetic differences that do exist between Mtb lineages, sublineages, and strains can have profound impacts on bacterial physiology and can influence virulence, immunogenicity, and drug resistance (Portevin et al., 2011; Carey et al., 2018). When measuring drug susceptibility profiles of clinical Mtb isolates, many groups have reported that clinical strains can have a wide range of minimum inhibitory concentrations (MIC) for some drugs (CRyPTIC Consortium, 2018; Farhat et al., 2019). Even strains that fall below the critical breakpoint for resistance can display a wide range of MIC values. Some of this MIC heterogeneity may be attributable to low-level acquired drug resistance mutations which are generally poorly understood (Wong et al., 2011; Colangeli et al., 2018; Hicks et al., 2020). However, MIC heterogeneity is also observed for new drugs with novel mechanisms of action and which have been used little (if at all) in the clinic (Bateson et al., 2022). Accordingly, barring unknown mechanisms of cross-resistance, there has been little or no selective pressure for the evolution of resistance towards these drugs.

There are at least two obvious explanations for MIC heterogeneity for new drugs. First, these differences could be explained by inter-strain differences in drug target vulnerability (Carey et al., 2018; Bosch et al., 2021). Using transposon sequencing, Carey et al. found differences in genetic essentiality between different Mtb isolates, with lineage 2 strains displaying a reduced reliance on the glyoxylate shunt (Carey et al., 2018). The authors could recapitulate this finding using a chemical inhibitor of malate synthase (GlcB), a key enzyme in this pathway. Further demonstrating this concept, recent work by Bosch et al. showed that the cytochrome C reductase gene, *qcrB*, displays enhanced genetic vulnerability in the lineage 2 strain, HN878, compared to the lineage 4 strain H37Rv (Bosch et al., 2021). Accordingly, HN878 is more susceptible to the QcrB inhibitor, Q203, than is H37Rv.

Alternatively, MIC heterogeneity may reflect differences in the levels of intrinsic drug resistance between Mtb strains. In cases where a particular strain is lacking an intrinsic resistance factor (i.e. *whiB7 or mmpL5*) or sustained mutations that result in its hyperactivitity, there can be a pronounced change in drug sensitivity with a clear genetic basis (Warit et al., 2015; Villellas et al., 2017; Merker et al., 2020; Li et al., 2022). However, in most cases it is difficult to pinpoint a genetic basis for the differences in intrinsic resistance levels between strains. Future studies aiming to map the genetic basis of intrinsic drug resistance could seek to define these mechanisms in representative Mtb clinical strains, beyond the most common lineage 4 reference strains H37Rv, Erdman, and CDC1551 (Borrell et al., 2019).

## Conclusions

TB remains one of the most difficult infectious diseases to treat. Even strains that are classified as drug sensitive display a remarkably high level of intrinsic resistance to many categories of drugs. Effective strategies must be developed not only to treat drug-resistant TB, but also to treat drug-sensitive TB in a shorter amount of time and with lower relapse rates. To do so, we must identify mechanisms of intrinsic drug resistance in Mtb and find ways around them. The cell envelope remains the most well-characterized and, perhaps, most important contributor to intrinsic drug resistance in Mtb. Successful disruption of the mAGP complex is an established method of disarming intrinsic resistance and sensitizing Mtb to antibiotics. In addition to the envelope, Mtb also encodes many other factors that can block antibiotic action once a drug has entered the cell. Future chemical-genetic studies will be paramount in furthering our understanding of the many layers of intrinsic drug resistance that Mtb has against a diverse set of antibiotics. A thorough genetic dissection of intrinsic resistance in Mtb will hopefully pave the way for more prioritized target-based drug discovery and medicinal chemistry efforts to develop faster-acting TB treatment regimens.

## Author contributions

Literature review: NP and JR, Writing and editing of manuscript: NP and JR. All authors contributed to the article and approved the submitted version.

## Funding

This work was supported by the Robertson Therapeutic Development Fund (JR), an NIH/NIAID New Innovator Award (1DP2AI14485001, JR), and a shared NIH TB research unit grant (U19AI162584, JR)

## Conflict of interest

The authors declare that the research was conducted in the absence of any commercial or financial relationships that could be construed as a potential conflict of interest.

## Publisher’s note

All claims expressed in this article are solely those of the authors and do not necessarily represent those of their affiliated organizations, or those of the publisher, the editors and the reviewers. Any product that may be evaluated in this article, or claim that may be made by its manufacturer, is not guaranteed or endorsed by the publisher.
